# Molecular Background of Toxic-Substances-Induced Morphological Alterations in the Umbilical Cord Vessels and Fetal Red Blood Cells

**DOI:** 10.3390/ijms232314673

**Published:** 2022-11-24

**Authors:** Szabolcs Zahorán, Ágnes Márton, Krisztina Dugmonits, Payal Chakraborty, Ali Khamit, Péter Hegyi, Hajnalka Orvos, Edit Hermesz

**Affiliations:** 1 Department of Biochemistry and Molecular Biology, Faculty of Science and Informatics, University of Szeged, H-6701 Szeged, Hungary; 2 Institute for Translational Medicine, Medical School, University of Pécs, H-7601 Pécs, Hungary; 3 Centre for Translational Medicine, Semmelweis University, H-1085 Budapest, Hungary; 4 Division of Pancreatic Diseases, Heart and Vascular Center, Semmelweis University, H-1085 Budapest, Hungary; 5 Department of Obstetrics and Gynecology, Albert Szent-Györgyi Medical School, University of Szeged, H-6701 Szeged, Hungary

**Keywords:** umbilical cord (UC), fetal red blood cells (RBCs), maternal smoking, ex vivo metal treatment, DNA double-strand break, matrix metallopeptidase-9/metallopeptidase inhibitor-1 (MMP-9/TIMP-1)

## Abstract

The relationship between smoking and human health has been investigated mostly in adults, despite the fact that the chemicals originating from sustained maternal smoking disrupt the carefully orchestrated regulatory cascades in the developing fetus. In this study, we followed molecular alterations in the umbilical cord (UC) vessels and fetal red blood cells (RBCs), which faithfully reflect the in vivo status of the fetus. We showed evidence for the decreased level of DNA-PKcs-positive nuclei in samples with smoking origin, which is associated with the impaired DNA repair system. Furthermore, we pointed out the altered ratio of MMP-9 metalloproteinase and its endogenous inhibitor TIMP-1, which might be a possible explanation for the morphological abnormalities in the UC vessels. The presented in vivo dataset emphasizes the higher vulnerability of the veins, as the primary target for the toxic materials unfiltered by the placenta. All these events become amplified by the functionally impaired fetal RBC population via a crosstalk mechanism between the vessel endothelium and the circulating RBCs. In our ex vivo approach, we looked for the molecular explanation of metal-exposure-induced alterations, where expressions of the selected genes were upregulated in the control group, while samples with smoking origin showed a lack of response, indicative of prior long-term in utero exposure.

## 1. Introduction

Smoking is one of the major risk factors for the development of cardiovascular diseases (CVDs), but it can also be associated with many other disorders, including chronic obstructive pulmonary disease, as well as tumors affecting all parts of the respiratory system. Tobacco smoke can be divided into two main parts: tar and the gaseous phase. The intake rate of harmful components during active smoking is 8% and 92% in favor of the gaseous phase. The two phases contain large portions of reactive free radicals (ROS) that are generally unstable with short half-life and high reactivity. The most significant representative of the tar phase is the quinone/hydroquinone complex, an active redox system capable of reducing the molecular oxygen (O_2_) to the superoxide anion (O_2_^•−^), which leads to the formation of hydrogen peroxide and a highly reactive hydroxyl radical (OH•). In the gaseous phase, the small oxygen- and carbon-centered organic radicals are also highly reactive due to their shorter lifetimes. The most significant representatives among them are the nitric oxide (NO), reactive olefins, and dienes [1].

The excessive prooxidant load that originates from the tobacco smoke alters the redox homeostasis either by direct and/or indirect effects, damaging proteins, nucleic acids, and lipids, which are mostly involved in the normal physiological functioning of the cells and tissues. The macromolecular damages are mostly associated with the ONOO^−^, formed by a spontaneous reaction with O_2_^•−^ and NO, which are highly diverse, e.g., double-strand DNA breaks, nitration, and/or oxidation of proteins and lipids [2,3,4,5,6].

In addition to the high pro-oxidant content, tobacco smoke also contains varying amounts and types of metals in their ionic form. The tobacco plant can absorb and accumulate toxic metals such as aluminum, cadmium (Cd), chromium, copper, lead, mercury, nickel, vanadium from the soil, and air pollutants, and hence, these will also be present in the tobacco smoke itself [7].

Cadmium, a heavy metal causing several adverse health effects in many organs, has been well classified as the No. 1 carcinogenic element for humans, because it can be directly linked to lung, kidney, and prostate cancer. Its indirect carcinogenic effect is based on competitive Zn^2+^ substitution at the tissue and cellular level (inhibition of DNA repair and antioxidant enzymes), induction of inflammatory responses, and increased production of ROS by the immune cells. Eventually, these effects may also influence the entire blood circulatory system, triggering the formation of CVD. Several studies suggest that CVD in smokers is strongly associated with elevated blood Cd^2+^/Zn^2+^ ratios. These patients are more likely to develop peripheral vascular disease, myocardial infarction, and hypertension [8,9,10,11,12,13].

The ways to combat or neutralize various stressors are at least as diverse as the damaging effects themselves. In living organisms, the effects of oxidants are counterbalanced by the antioxidant family members. Prominent candidates acting as non-enzymatic antioxidants are the metallothioneins (MTs), the low molecular-weight, cysteine-rich molecules. Their main task is to balance the metal household of the cells and tissues, but they also have a significant free radical-neutralizing capacity along with their inhibitory effect on the activity of ROS-producing enzymes, such as superoxide dismutase and inducible nitric oxide synthase (NOS2). Different isoforms have been identified in the MT-I family, the most important ones for the cardiovascular system and endothelial cells being MT1E, MT2A, and MT3 [14]. Their promoter region contains several stimulatory response elements, such as metal, glucocorticoid, and antioxidant regulatory elements, which may be associated with the inflammatory mediators. Thus, their expression can be easily induced by increased ROS or by metal ions such as Cd^2+^ and lead [15].

The cell response to endogenous and/or exogenous stimuli is important at any time of life. In the case of the developing fetus, it is even more critical to reserve cell integrity, nucleic acids structure, and homeostatic balance. One of the most important dangers to the developing fetus is the long-term exposure to oxidative/nitrosative stress, which becomes directly or indirectly triggered by the influence of toxic components originating from maternal smoking. The primary targets for such stressors are the circulating fetal red blood cell (RBC) population and the umbilical cord (UC) vein, carrying O_2_-rich blood from the placenta to the fetus along with other, unfiltered harmful substances.

Based on our earlier studies, the endothelium ultrastructure of UC vessels originating from neonates born to smoking mothers showed significant differences to the non-smoker’s healthy origin. Following such typical changes are the fragmentation of the nucleus, loosened cell–cell contacts, and detachment from the basal membrane [16]. Disruptions in the processes of intercellular connections, which also means the disappearance of organized tissue structures, are mainly orchestrated by matrix metalloproteinases (MMPs) and their tissue inhibitors (TIMPs). Any alteration in the delicate balance in their expression level induces both structural and functional changes of the endothelium [17,18,19,20]. Furthermore, in the samples with smoking origin, significant increases were measured in the apoptotic cells; around 65% of the isolated vein endothelial cells were either in the early or late apoptotic phase. Despite the massive cell death, the UC vessels were still functional and showed cellular response to an ex vivo stress condition [16].

Apart from direct exposure to the harmful materials, it is assumed that there exists an intimate crosstalk between the vascular endothelium and the fetal RBCs, where the endothelium becomes affected by the altered protein profile in the RBCs [21]. Previously, we pointed out that maternal smoking induces morphological aberrations and functional alterations in the fetal RBC population, which makes it likely that the endothelial nitric-oxide-synthase (NOS3)-dependent NO production by RBCs is not available as a compensatory mechanism in the case of endothelial dysfunction [22,23]. In fact, the NO production is impaired by RBC-NOS3 and the Arginase-1 expression is increased [21]. The status of fetal RBCs and UC vessels reflects the impact of substances, originating from an improper maternal lifestyle. The detectable changes in the RBC populations and UC vessels may serve as markers of the damages that have been imposed on the developing fetus or might be associated with the development of other diseases in post-natal or later life.

General consequences of fetal oxidative stress lie with the set of adaptive processes that involve a constant change in the structure, physiology, and metabolism of the fetus life after birth. These processes are tightly regulated and any alteration in them can affect the in utero development at many levels, which may predispose to many diseases in their later life (even in adulthood). Maternal smoking-induced oxidative stress affects the pattern of gene expression at all levels of regulation [24,25,26,27].

In this study, we investigated the impact of maternal smoking on the fetal gene expression. Parallel to that, we attempted to model the toxic-exposure-induced alterations by an ex vivo Cd^2+^ treatment on the fetal RBCs and isolated UC vein with both non-smoking (NS) and smoking (S) origins.

## 2. Results

### 2.1. Molecular Background of the Ultrastructural Damages in the Umbilical Cord Vessels

#### 2.1.1. DNA Repair System

The main feature of DNA damage as induced by the harmful effects of free radicals and heavy metals is the appearance of a single- or double-strand breaks, which are counteracted by repair mechanisms. In this work, we analyzed the nuclear localization of the phosphorylated ɤH2A.X and the phosphorylated DNA-dependent protein kinase catalytic subunit (DNA-PKcs), involved in the double-strand break response, by double immunolabelling on UC sections.

**Figure 1:**
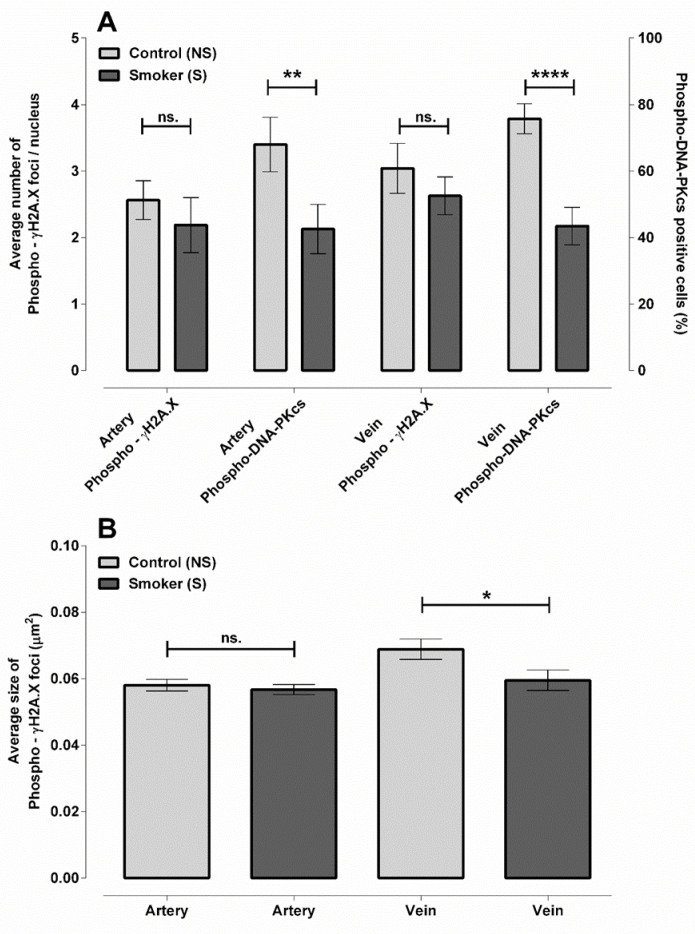
Analysis of DNA double-strand breaks and the efficiency of DNA repair in NS and S UC vessels. Graphical representation showing the quantitative analysis of phospho-ɤH2A.X, phospho-DNA-PKcs (**A**) specific immunolabelling, and ɤH2A.X focal mean size (**B**). Quantified regions and number of samples: NS (n = 101 arteries and 198 venous endothelial cell nuclei from 3 samples); S (n = 169 arteries and 189 venous endothelial cell nuclei from 3 samples). Statistical analysis was conducted by using two-way (**A**) and one-way (**B**) ANOVA followed by Holm–Sidak’s multiple comparisons test (ns. = not significant; * *p* ≤ 0.05; ** *p* ≤ 0.01; **** *p* ≤ 0.0001).

Based on the immunohistochemical analysis of the endothelial cell nuclei, there was no significant difference in the average quantity of ɤH2A.X foci within the NS and S sample groups, neither on arterial nor on the venous level. In parallel with this, we found that the number of phosphorylated DNA-PKcs-positive cells, indicative of the activation of the non-homologous end-joining (NHEJ) repair pathway, showed a significant (25% and 30%) difference between the NS and S arteries and the veins, respectively (Figure 1A). These data suggest that with the similar frequency of DNA double-strand breaks, the compensatory activation of the repair pathways is not proper. However, even considering our technical limitations, with simple particle analysis, we aimed to estimate the average size of the ɤH2A.X foci, which basically determines the proper assembly of the repair apparatus. In this, we found a significantly lower foci size in the venous nuclei of the S populations (Figure 1B).

#### 2.1.2. Matrix Metalloproteinase and Their Tissue Inhibitors

The molecular background of the loosened endothelial cell–cell connections was investigated by the measurement of MMP-9 and MMP-2 levels, which catalyze the extracellular matrix (ECM) degradation processes, along with TIMP-1 and TIMP-2 proteins, involved in their specific inhibition. Consecutive sections from identical samples were immunolabelled either with anti-MMP or anti-TIMP antibodies and quantified in the endothelial layer of the arteries and veins of NS (n = 4) and S (n = 4) samples.

**Figure 2:**
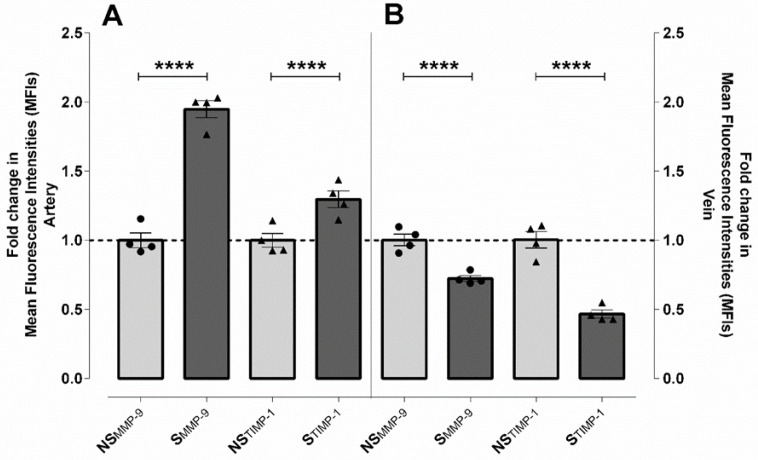
Expression of MMP-9 and its inhibitor, TIMP-1, in the endothelial layer of UC vessels with NS and S origins. Graphical representation of the immunohistochemical analysis showing the differences in mean fluorescence intensities (MFIs) between the arteries (**A**) and veins (**B**) of the NS and S samples (mean ± SEM). Each point on the graph indicates independent samples; NS-MMP-9 (n = 144 arteries and 35 venous regions of interest from 4 individual samples); S-MMP-9 (n = 245 arteries and 26 venous regions of interest from 4 independent samples). NS-TIMP-1 (n = 430 arteries and 54 venous regions of regions from 4 independent samples); S-TIMP-1 (n = 424 arteries and 32 venous regions of interest from 4 independent samples). Statistical analysis was performed by using an unpaired *t*-test followed by a Mann–Whitney test to compare ranks (**** *p* ≤ 0.0001).

**Figure 3:**
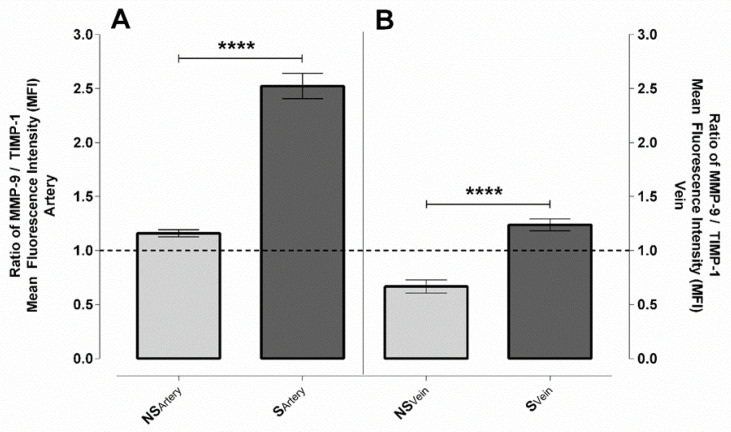
Ratio of MMP-9/TIMP-1 in UC samples with NS and S origins. Graphical representation of the mean fluorescence intensity (MFI) ratios from the immunolabelled arteries (**A**) and veins (**B**) (mean ± SEM). Independently quantified regions and samples: NS-MMP-9 and NS-TIMP-1 (n = 129 arteries and 26 venous regions of interest from 4 independent samples); S-MMP-9 and S-TIMP-1 (n = 144 arteries and 26 venous regions of interest from 4 independent samples). Statistical analysis was performed by using an unpaired *t*-test followed by a Mann–Whitney test to compare ranks on the raw dataset (**** *p* ≤ 0.0001).

In the UC arteries with smoking origin, both the MMP-9 and TIMP-1 levels were increased ~2 and ~1.3-fold, respectively (Figure 2A and Figure S1). Considering the same sample sets, both protein levels were somewhat lower in the veins of the S samples compared to the NS ones. The extent of difference was greater in TIMP-1 in comparison to MMP-9 (~0.7- and 0.5-fold, respectively) (Figure 2B and Figure S1). In addition to the absolute values, the ratio of the two proteins significantly determines the fate of cells/tissues as TIMP-1 binds to MMP-9 in a 1:1 stoichiometric ratio. Based on our datasets, in the control arteries, the ratio of MMP-9/TIMP-1 was ~1, and the effect of the proteins was well balanced. In the veins, TIMP-1 dominated the system, with the ratio of MMP-9/TIMP-1 being ∼0.6. In contrast to the samples with S origins, the regulation was driven by MMP-9; the ratio of MMP-9/TIMP-1 was ~2.5 in the arteries and ~1.3 in the veins (Figure 3A,B). In these systems, the expression of MMP-2 and TIMP-2 was at the limit of detectability.

### 2.2. Ex Vivo Cd^2+^-Exposure-Induced Alterations

#### 2.2.1. Expression of *mmp-9* and *timp-1* Genes

**Figure 4:**
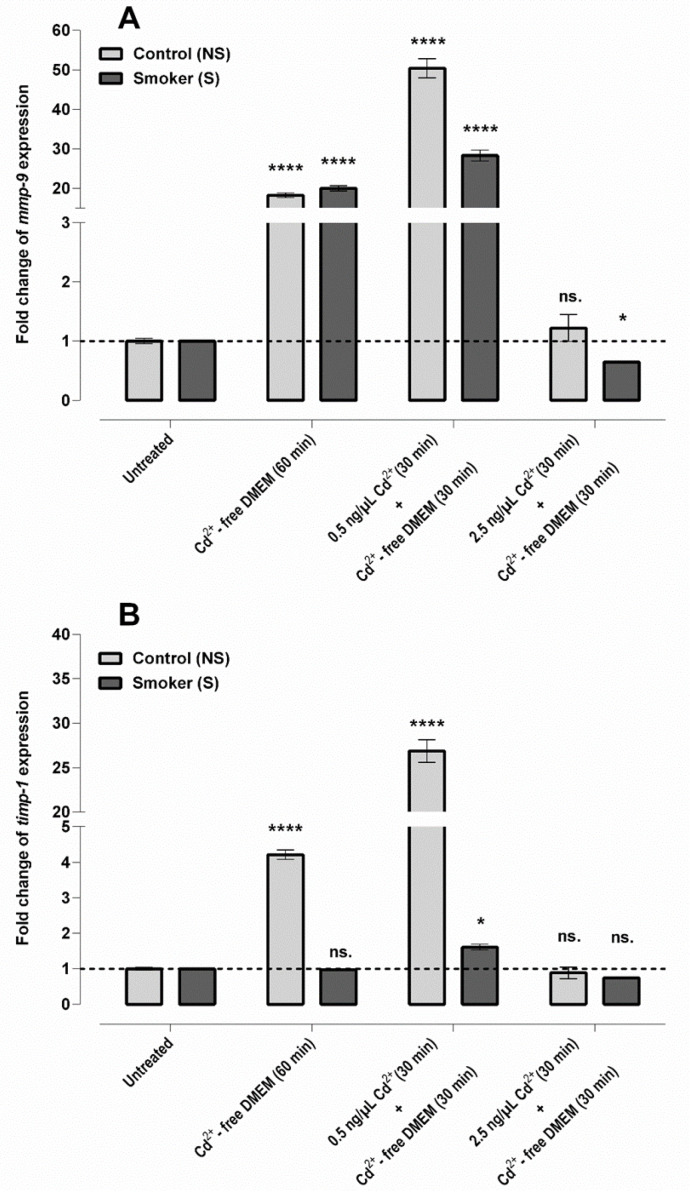
Stress-induced alteration in *mmp-9* and *timp-1* expression in an ex vivo model system: *mmp-9* (**A**) and *timp-1* (**B**) expression relative to untreated samples induced by heat shock and combined (heat + Cd^2+^) stress. Number of items: control (n = 3), smoker (n = 3); all results conducted with 3 parallel reactions (mean ± SEM). Statistical analysis was conducted by using two-way ANOVA and the Holm–Sidak post hoc test (ns. = not significant; * *p* ≤ 0.05; **** *p* ≤ 0.0001).

To test the transcriptional activity of the endothelium under stress conditions, fragments of veins from both origins were isolated and samples were incubated at 37 °C with and without Cd^2+^ at concentrations of 0.5 and 2.5 ng/μL. The transcriptional patterns of *mmp-9* and *timp-1* were followed by quantitative RT-PCR (for primer sequences, see Table 1). The basal expression of both genes was significantly lower in the samples with smoking origin compared to the control (16- and 5-fold, respectively, Figure S2). Stress-induced alterations in the transcriptional patterns showed that *mmp-9* was highly induced by heat shock and was combined with heat and Cd^2+^ treatment. In the case of heat treatment, the response of NS and S samples was nearly identical, a ~20-fold increase, while in the case of the combination treatment, the induction of *mmp-9* in the NS samples was almost twice as high as that measured in the S samples, 50- and 28-fold, respectively (Figure 4A). For *timp-1*, we found that only control samples responded to the treatments, with no significant change in veins with smoking origin. *timp-1* induction was ~4-fold for heat shock and nearly ~27-fold for the combined heat and Cd^2+^ concentration of 0.5 ng/μL. Cd^2+^ at a concentration of 2.5 ng/μL did not induce transcription of the examined genes in either of the two groups (Figure 4B).

#### 2.2.2. Expression of Metallothionein Genes

The immediate effect of short-term Cd^2+^ exposure on the expression of 3 mt genes, *mt1e*, *mt2a,* and *mt3* (for primer sequences, see Table 1), was investigated, specifically associated with the defense pathway against heavy metal exposure in the vascular endothelial cells. The basal expression of the three selected genes differed significantly between NS and S samples. The mRNA levels of *mt1e* and *mt2a* were higher in the control samples at 3- and 1.5-fold respectively, while that in the case of *mt3* was lower, ~7 fold (Figure S3)

**Figure 5:**
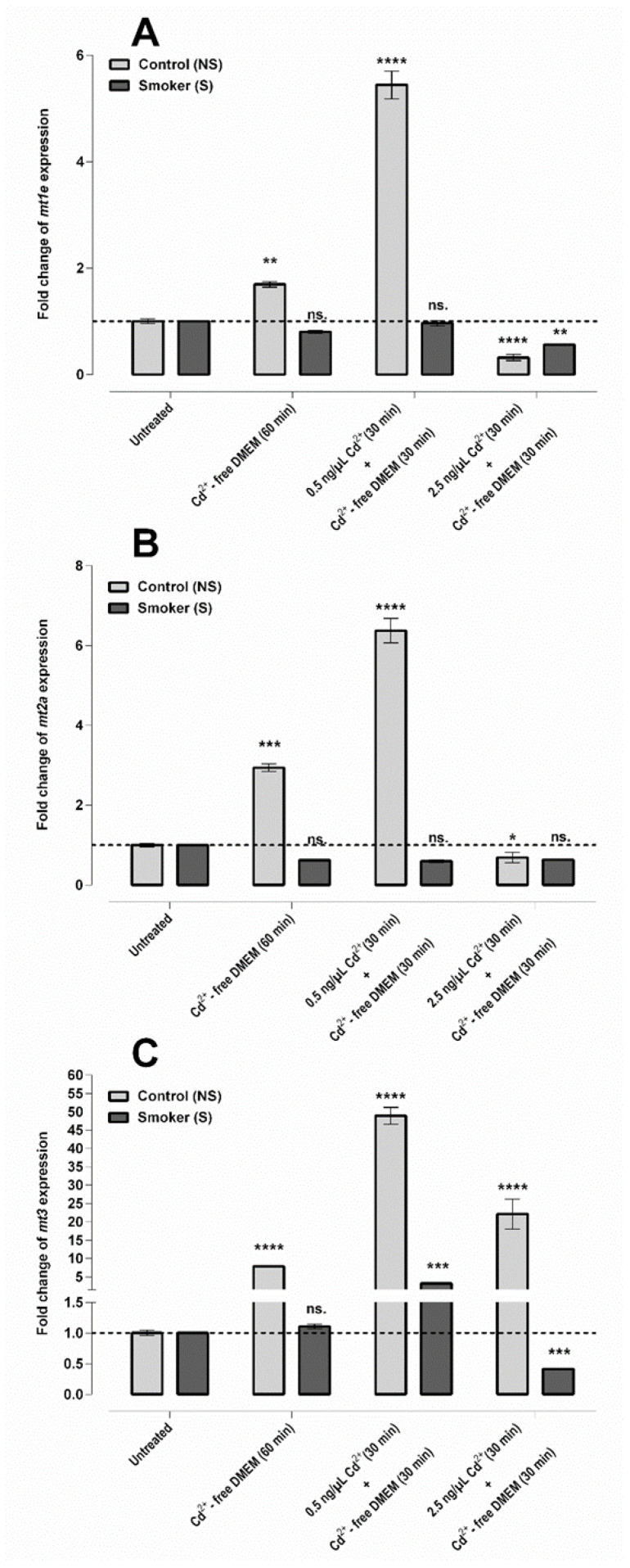
Ex vivo treatment of isolated UC vein samples. Expression levels of *mt1e* (**A**), *mt2a* (**B**), and *mt3* (**C**) genes in NS and S vein samples. Three independent samples were selected from both groups and quantitative RT-PCR reactions were accomplished in triplicate (mean ± SEM). Statistical analysis was conducted by using one-way ANOVA and Tukey’s multiple comparisons test on the ΔCt values (ns. = not significant; * *p* ≤ 0.05; ** *p* ≤ 0.01; *** *p* ≤ 0.001; **** *p* ≤ 0.0001).

In the control group, *mt1e* gene expression was slightly induced by heat treatment (~1.7-fold), while under the influence of combined exposure: heat and Cd^2+^ at a concentration of 0.5 ng/μL, the increase in mRNA level was ~5.4-fold. In the S group, *mt1e* transcription did not exhibit a significant change, neither by heat nor with the combined exposure. Cd^2+^ at higher concentration, 2.5 ng/μL, repressed *mt1e* gene expression in both groups (Figure 5A).

The expression of the *mt2a* gene compared to the untreated NS samples was increased by ~3-fold for heat and ~6.3-fold for combined heat and 0.5 ng/μL Cd^2+^ treatment, while no significant changes were observed using an elevated Cd^2+^ concentration (2.5 ng/μL). S samples showed no significant difference in the *mt2a* mRNA level, after any of the treatments (Figure 5B).

Heat shock and a lower concentration of Cd^2+^ in combination induced *mt3* expression in the control group, ~8- and ~49-fold, respectively. In parallel, the effect of high Cd^2+^ concentration (2.5 ng/μL) in combination treatment was somewhat lower, where a ~22-fold increase was measured. In the case of S samples, heat treatment had no effect (~1.1-fold), while the Cd^2+^ concentration of 0.5 ng/μL induced the gene expression with ~3.2-fold, in comparison to the untreated group (Figure 5C). High Cd^2+^ concentration (2.5 ng/μL) repressed the mRNA level in the S samples.

#### 2.2.3. Red Blood Cells

Effect of Short-Term Cd^2+^ Treatment on Red Blood Cells with Smoking and Non-Smoking Origins

In our earlier publication, we presented that the maternal smoking-induced morphological aberrations in the fetal RBC population could be mimicked by Cd^2+^ treatment of the control cell population in an ex vivo experiment [22].

Total blood from both origins was incubated with and without Cd^2+^ at a concentration of 0.5 ng/μL for 1 h at 37 °C and followed the activation of the Caspase-3 enzyme and the accumulation of 4-hydroxynonenal (4-HNE), indicative of membrane damage. Quantitative measurements were performed by fluorescence-activated cell sorting (FACS) analysis on double immunolabelled anti-active Caspase-3/Glycophorin and anti-4-HNE/Glycophorin RBC populations, where, in each case, we found low and high intensity levels.

**Figure 6:**
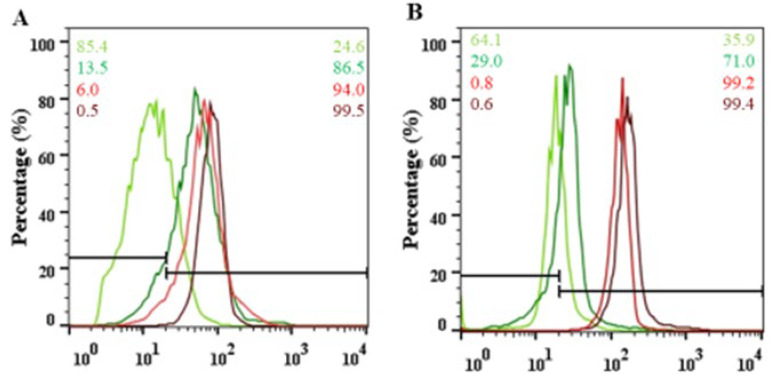
Representative histograms based on FACS (fluorescence-activated cell sorting) analysis on isolated arterial cord RBC-NS (green) and RBC-S (red) populations with (dark green and red) and without (pale green and red line) exposure to Cd^2+^ at a concentration of 0.5 ng/μL for 1 h. Quantification of the accumulation of active Caspase-3 (**A**) and the extent of macromolecular damages as followed with the lipid peroxidation marker 4-HNE (**B**) was carried out in glycophorin-positive cell populations. An arbitrary borderline was considered dividing the total RBC population into basal and high-intensity cells.

Detailed FACS analysis revealed a significant difference in Caspase-3 activation between the two untreated sample groups. The active enzyme level was nearly 90% higher in the RBC-S population than in the RBC-NS. After the Cd^2+^ treatment, the enzyme activity increased to ~80% in the RBC-NS, while in the RBC-S samples, the elevation was maximized to ~20% (Figure 6A).

Similarly, in the level of membrane damage, there was a significant difference in the untreated sample groups. In the NS population, only 30% of the cells showed high 4-HNE level, while in the smoking group, 100% of the cells were affected. The Cd^2+^ treatment increased 4-HNE accumulation in 70% of the NS cells, while in the S population, the rise was only 15% (Figure 6B).

## 3. Discussion

Maternal smoking significantly contributes to ROS production/accumulation in the placenta, circulating fetal RBCs, and the endothelial layer of the UC vessels, which directly and/or indirectly affects the fetal development. Feltes and colleagues recently published data analysis on a large number of in-depth and systemic studies based on their chemobiological relationships between nicotine and 50 other tobacco-related smoke constituents. The authors pointed out that such potent hazardous substances influence molecular pathways orchestrating both the embryonic and fetal development [24]. Impairment of these programmed processes (e.g., intercellular communication and signaling, hormone synthesis, metabolism, DNA repair, and inflammatory responses) occurs at their pivotal steps during the intrauterine development that might also become associated with the long-lasting functional changes. The proper functioning of RBCs and blood vessels in the UC is a prerequisite for satisfactory nutrition and oxygen supply to the fetus. Thus, any damage to the RBC population and the UC vessels can directly affect the intrauterine development. Furthermore, any morphological and functional alteration in these systems can be interpreted as an imprint of damage to the fetus. Datasets presented in this study and even in our earlier work [16] clearly suggest that sustained maternal smoking during pregnancy induces morphological and functional damage in the fetal RBC population and in the UC vessels. The fact is that not only are the primarily exposed UC veins affected, but even the arteries, which might indicate an extensive, systemic loss of function.

Based on ultrastructural changes in the endothelium of S vessels (highly condensed chromatin, electron-dense pattern, and lobular nucleus marking their early stage in the process of fragmentation) [16], we hypothesized that the damage also becomes reflected in their DNA integrity and repair system. The DNA double-strand break is the most well-known threat for the genomic integrity. The ɤH2A.X foci are not only markers of the double-strand breaks, but basically determine the position for the assembly of the recruited proteins in the repair system, while the level of the phospho-DNA-PKcs may be related to the success of the repair mechanism [28,29,30]. For the repair, two main possibilities are proposed: one of them is the homologous recombination (considered as S- or G2-phase-specific) and the other one is the NHEJ pathway, particularly in the G0/G1 phase of the cell cycle. In the latter case, the presence of active, phosphorylated DNA-PKcs is a prerequisite for successful error repair [31,32]. DNA double-strand breaks were detectable in the endothelial cells of both the NS and S vessels, but the proportion of active phospho-DNA-PKcs, specialized for the NHEJ repair, was significantly lower due to maternal smoking. One of the reasons behind this phenotype could be the reduced activity of their repair mechanisms in the S samples, especially in the vein, where the number of phosphorylated DNA-PKcs-positive nuclei was extremely low (40%), suggesting an impaired assembly of the repair apparatus.

There were also other striking distinctions between these two types of sample population, such as the loosened endothelial cell–cell contact and their detachment from the basal membrane [16]. In fact, tissue rearrangement plays a key role in the process of ontogenesis and, further, its precise control is crucial for the tissue fate. MMPs and their inhibitors, TIMPs, have a fundamental role in the remodeling of the ECM, and its subsequent ultrastructural alterations may be a consequence of an imbalance in the MMP-TIMP system. The ratio of MMP/TIMP regulates the dynamics of ECM, not only in normal but even under pathological conditions, as with the CVDs, including atherosclerosis, cardiomyopathy, and myocardial repair following infarction, which can serve as markers of these disorders. Oxidative stress, which is involved in CVD, can also stimulate MMP production and its activation [17,18,19,20,33].

MMP-9 and MMP-2 as well as TIMP-1 and TIMP-2 were examined in the control and smoker groups [34,35]. Levels of MMP-2 and TIMP-2 in both vessel types were around the detection limit with no difference between the two sample populations. Based on this result, we hypothesize that the role of MMP-2 in the UC system is most significant at the early stage, during the formation of the UC vessels. In the case of the MMP-2/TIMP-2 pair, it has been previously observed that their levels are positively correlated with the processes of angiogenesis and neovascularization, and these processes are not characteristic of the UC endothelial cells [36,37]. In parallel, the MMP-9 and TIMP-1 expression showed origin- and vessel-specific characteristics; a significant increase was measured for both proteins in the arteries of S samples, while in the veins, significant down-regulation was detected. Though the elevated MMP-9 level does not necessarily mean increased MMP-9 activity, an up-regulation in the gene expression indicates an alteration in the cellular homeostasis and predicts a less beneficial cell condition, which may lead to the observed ultrastructural changes. The decrease in both protein levels in the vein might be a consequence of a general downregulation in the transcriptional activity and/or protein degradation in the S system, due to the long-drawn stress stimuli. A significant increase was measured for both proteins in the artery of S samples, resulting in a more than 2-fold dominance of MMP-9. Based on this value, it is clear that in the arteries, the increased MMP-9 expression cannot be counteracted by TIMP-1, which may lead to the observed phenotypic differences in their ultrastructure between the sample groups. This supposition seems to be correct, not only due to the close relationship between the two proteins but also due to the stoichiometric ratio of the proteins in the complexes that formed during inhibition, which is 1:1 [38]. In the S veins, both gene expressions were downregulated with a different ratio of MMP-9/TIMP-1, and it was around 1.5. Thus, the protease activity dominated the process, leading to massive tissue damage. The decreased level of both proteins may be due to a general decrease in the transcriptional activity of the S system. Indeed, our transcriptional studies clearly indicated that the baseline expression of both genes were significantly lower than in the NS samples. Regarding the complex transcriptional regulation of both genes, the reduced protein levels might be a direct consequence of the low mRNA values [39].

The possible modulatory effect of NO on *mmp-9* and *timp-1* at the mRNA levels is documented in the literature. Although there are some contradictions in the published dataset, it seems likely that the increased exo- or endogenous NO level downregulates the *mmp-9* and *timp-1* expressions, and vice versa. Inhibition of NOS3 activation strongly increases the expression of both genes [39,40,41]. In our previous publication, we demonstrated a strong downregulation of NOS3 and its activation by phosphorylation at the Ser 1177 position both in the UC arteries and veins with smoking origin [16]. This observation highly correlates with the increased MMP-9 and TIMP-1 protein level in the arteries. However, the veins of S samples show significant downregulation both at the mRNA and protein levels, which indicates an elevated NO production in this system. Expectedly, along with the impaired NOS3 activation, in order to maintain adequate vascular tone, the upregulation of NOS2, as a rescue mechanism, along with a significantly elevated nitrite/nitrate pool and activation of the XOR system was well detected [16]. As the catalytic activity of NOS2 is 100-1000 fold than that of NOS3 [42], a highly elevated NO production and peroxynitrite accumulation mark the system with smoking origin. The high level of reactive nitrogen species seems to overwrite the inductive effect of mild Cd^2+^ accumulation, remaining unfiltered by the placenta, on MMP-9 expression in the S samples. An acute or chronic Cd^2+^ exposure increases *mmp-9* expression in the human endothelial, prostate epithelial, and malignant monocyte cell cultures, and also in the rat heart tissues [43,44,45].

The presented molecular dataset along with the alterations in the ultrastructure as observed by the transmission electron microscopy clearly indicated an impaired endothelial function due to maternal smoking and pointed out the vein as the prime target with an increased and direct exposure to toxic substances. However, despite all the molecular and morphological alterations, the system still remains functional. In an ex vivo setup, we tested the responsiveness of S-derived UC veins and proved that the transcriptional machinery can be activated by direct/indirect exposition to stressors; the upregulation of the *mmp-9* expression by heat treatment was comparable to the NS values. However, while NS samples showed extra sensitivity to Cd^2+^ exposure (a *mmp-9* expression more than doubled in the presence of Cd^2+^, 50×, compared to heat treatment 20×), contrastingly, samples with smoking origin had null or a slight response to Cd^2+^ in the medium. This supports the fact that in the case of control veins, the ex vivo Cd^2+^ acts as a novel stressor, whereas the S system may have already been exposed to heavy metals during pregnancy; hence, their response would be less pronounced.

In the S samples, similar to the *mmp* and *timp* genes, low basal expression was found for the examined *mt*s, except for *mt3*, further supporting reduced transcriptional activity in this system. The MT1E and MT2A isoforms are expressed in many tissues and are inducible by oxidative stress or even low concentrations of heavy metals. For MT3, it is important to note that this isoform has appeared for decades as a central nervous system and testis-specific, non-inducible MT-isoform [46]. In 2014, Schulkens et al. demonstrated the in vitro presence of *mt3* mRNA in HUVECs [14]; however, the in vivo behavior and their role in the cardiovascular system were still unknown. Based on recent publications, MT3 is partially inducible, depending on the heavy metal concentration [47,48,49]. It is most likely that the predominant MT1 and MT2 isoforms have been induced at the transcriptional level in the endothelial cells of S samples at the early stages of pregnancy and contributes to the development of heavy metal tolerance. The MT3, with higher metal-sensitivity, appears only at the later stages of development with the progress of Cd^2+^ accumulation. The high *mt3* mRNA baseline level in the S samples (7-fold the NS) seems to be clear evidence of it. These data are also supported by the ex vivo Cd^2+^-treatment-induced transcriptional activation. In the case of NS samples, the lower Cd^2+^ concentration strongly induces the mRNA level of all three *mt* genes. Furthermore, *mt3* expression was induced by Cd^2+^ at even higher concentration (2.5 ng/µL), which was not seen in the case of any other investigated gene. In the case of S samples, genes encoding the three MT isoforms were non- or slightly (*mt3*) inducible, upon Cd^2+^ exposure, which clearly pointed out that the adaptation to Cd^2+^ occurred during pregnancy due to previous heavy metal exposures.

Overall, it is clear from this study that maternal smoking induced specific changes in the endothelium of UC vessels, and the subsequent alterations are connected to the prior metallic exposure, as samples with smoking origin are no longer or less responsive to an ex vivo Cd^2+^ treatment.

We also came to a similar conclusion by testing the fetal RBCs, isolated from the S population. The ex vivo Cd^2+^ treatments did not affect the studied parameters in this sample group; however, it should be emphasized that the basal expression of the measured parameters was significantly higher in the untreated S samples compared to controls, indicating the in vivo metal exposure during fetal development. As there is a continuous presence of toxic material during pregnancy, it is most likely that the RBCs adapt to this condition with a modulated gene expression pattern.

In our previous work, we pointed out that as a result of maternal smoking, fetal RBCs become a source of oxidizing agents, which are most likely one of the reasons of altered gene expression and cell viability. However, the modified status of fetal RBCs also has a fundamental effect on the vascular conditions. Based on a recent publication, there is an active communication between the vessel endothelium and RBCs, where any functional alterations in the RBCs have an impact on the endothelial status [21]. One of the possible intermediary agents in the circulated RBCs could be the accumulated peroxynitrite, a highly reactive nitrogen species, formed spontaneously from O_2_^•−^ and NO [50]. Peroxynitrite-dependent cytotoxicity on the one hand relies on its ability to trigger lipid peroxidation in membranes, resulting in an increased level of reactive aldehyde, 4-HNE. 4-HNE can be considered a reliable biomarker for vascular complications in various pathological processes. On the other hand, Pernow and his coworkers also provided evidence that the RBCs-produced peroxynitrite is involved in the upregulation of arginase, not only in the RBCs but also in the vessel endothelium. Endothelial arginase, in turn, acts as an intermediary molecule for the development of endothelial dysfunction [51]. In our previous work, we showed that arginase expression is increased in the endothelial layer of both the veins and arteries in S samples, in parallel with decreased NOS3 expression and its activating phosphorylation [16]. The exact way of peroxynitrite-mediated signaling between RBCs and endothelial cells is still unclear, but it is most likely the peroxynitrite-induced lipid peroxidation that convincingly takes part in this process.

## 4. Materials and Methods

### 4.1. Sample Collection

UC and total blood samples were collected from neonates born to heavy-smoking mothers (at least 10 cigarettes per day during the entire pregnancy period) (nS = 12), along with samples from the age-matched neonates born to non-smoking mothers (nNS = 14). At the Department of Obstetrics and Gynecology, University of Szeged, Hungary, in compliance with the rules and regulations of the Declaration of Helsinki and as per the approved study protocol by the Ethics Committee of the University of Szeged (16/2016), was the study conducted. Maternal age below 18 years, gestational age below 37 weeks, infection, gestational diabetes, high blood pressure, stroke, atherosclerosis, heart failure, under medication, and intrauterine distress were excluded from the sample collection. The nutritional status during pregnancy was satisfactory and no cases of malnutrition or evidence for genetic disorders were reported. Samples were collected and processed for further use immediately after delivery.

### 4.2. Immunolabeling, Fluorescence-Activated Cell Sorting (FACS), Imaging, and Data Analysis

RBCs were isolated from total blood by centrifugation at 500× *g* for 10 min at 4 °C. The lower two-thirds of the RBC phase was washed 3 times using isotonic saline solution (0.9% NaCl) and fixed with 4% (*w*/*v*) paraformaldehyde in 0.05 M phosphate buffer (PB, pH = 7.4) for 30 min. After extensive washing with PB, cells were permeabilized using 0.1% Triton X-100 for 20 min at room temperature (RT), followed by 3 times of washing with PB. To block nonspecific binding of the primary antibodies, a treatment of PB containing 4% bovine serum albumin and 5% normal goat serum was used for 2 h at RT. RBCs were immunolabelled with the primary antibodies (Table 2) in PB containing 1% bovine serum albumin and 5% normal goat serum at 4 °C overnight, followed by washing and incubation with Alexa^®^647 and/or Alexa^®^488 conjugated secondary antibodies for 2 h at RT. After washing, RBCs were processed for quantitative analysis (FACS, BD FACSCalibur™, BD Biosciences, Franklin Lakes, NJ, USA) and data were analyzed by FlowJo. (FlowJo. Software for Windows Version 10, BD Biosciences).

For the UC samples, 2–3 cm whole tissue pieces were fixed with 4% (*w*/*v*) paraformaldehyde in PB, and cryoprotected with 30% (*w*/*v*) sucrose in PB supplemented with 0.1% (*w*/*v*) sodium-azide. The specimens were embedded in Tissue-Tek^®^ O.C.T (Sakura Europe, Alphen aan den Rijn, Netherlands), and cryo-sectioned (16 µm) on Superfrost™ ultra plus^®^ (J3800AMNZ) slides from Thermo Scientific (Waltham, MA, USA). The immunolabeling was carried out as mentioned above. Finally, the sections were examined with a ZEISS LSM 880 confocal laser scanning microscope, equipped with an Axiocam 503 mono (Carl Zeiss Microscopy GmbH, Jena, Germany) and Zen 2.1 software for image acquisition. Arteries and veins of each UC section were captured in at least 3–5 independent fields of view with the following parameters: 8-bit images, Plan-Apochromat 40×/1.4 Oil DIC M27 objective; excitation with Argon (488 nm) laser line for Alexa 488 fluorophore, Helium-Neon (633 nm) laser line for Alexa 647 fluorophore, violet (405 nm) laser line for DAPI; detection filters: 493–591 nm, 630–755 nm, and 410–479 nm; pinhole = 1 airy unit. The raw images were semi-quantified with ImageJ 1.50i software (NIH, USA), following the previously published description [16,52]. The results were summarized in MS^®^ Excel, and the changes in mean fluorescence intensity (MFI) were reported relative to the control values.

### 4.3. Ex Vivo Experiments

Total blood of independent individuals (NS = 14, S = 12) was subjected to Cd^2+^ treatment for the ex vivo experiments. Samples were incubated with and without cadmium acetate Cd(CH_3_COO)_2_ × 2H_2_O in 0.9% NaCl solution at the final concentration of 0.5 ng/μL of Cd^2+^ for 60 min at 37 °C. After treatment, RBCs were isolated and processed for FACS analyses.

The UCs were processed as previously described [16]. In short, the UC veins from independent individuals (NS = 3, S = 3) were isolated on ice, within 1 h after delivery, and were distributed for short, equal pieces (2–3 cm). For each sample, one piece was used as an absolute control, which was immediately frozen in liquid nitrogen and stored at −80 °C until the further steps. In order to model acute heavy metal stress, the fragments were transferred into 12-well plates, after a short period of acclimatization at room temperature. The plates contained Dulbecco’s modified Eagle’s medium (with 4.5 g/L of glucose and with l-glutamine, DMEM, Lonza Bioscience, Basel, Switzerland: 12-604F) with and without 0.5 ng/µL and 2.5 ng/µL of Cd^2+^ (Sigma–Aldrich, St. Louis, MO, USA: 289159). The Cd^2+^ exposure was carried out at 37 °C with 5% CO_2_, for 30 min. After that, fragments were rinsed thoroughly with pre-warmed DMEM two times and further incubated for 30 min in a Cd^2+^-free DMEM. Considering the possible effects of heat shock on gene expression, fragments were incubated in Cd^2+^-free DMEM solution in parallel with the metal exposure. Finally, all fragments were frozen in liquid nitrogen and kept at −80 °C until RNA extraction.

### 4.4. Total RNA Extraction, First-Strand cDNA Synthesis, and Real-Time Quantitative PCR (RT-qPCR)

Blood vessels originating from the ex vivo experiments were powdered in liquid nitrogen and homogenized in TRI reagent (Zymo Research, Irvine, CA, USA), followed by chloroform extraction and centrifugation (room temperature, 17,000× *g*, 20 min). The total RNA content of the supernatant was extracted using the Direct-Zol RNA kit (Zymo Research) according to the manufacturer’s instructions. The purity and concentration of the RNA solution were determined with a NanoDrop ND-1000 spectrophotometer (Thermo Scientific). Reverse transcriptions were performed according to the instructions of the Maxima H Minus First-Strand cDNA Synthesis Kit (Thermo Scientific), and 500 ng of total RNA and 100 pmol oligo(dT)18 primer were incubated for 5 min at 65 °C, followed by 30 min at 60 °C, in a final volume of 20 µL; the reaction was terminated with 5 min of incubation at 85 °C. Real-time qPCR was performed using a Luminaris Color HiGreen Low ROX qPCR Master Mix (Thermo Scientific, United States) in an Applied Biosystems 7500 Real-Time PCR System (Life Technologies, Budapest, Hungary) at the following program: 95 °C for 10 min, followed by 40/45 cycles of 15 s at 95 °C and 60 s at 60 °C. The RT-qPCR reactions for each sample were performed in triplicate to increase the reliability of the measurements. The threshold values (Ct) were normalized to the internal control 18S rRNA and the increase in the mRNA levels was calculated using the ΔΔCt method [53].

### 4.5. Data Presentation

Data analyses and graphical representation were performed with GraphPad Prism version 6.00 (GraphPad Software, La Jolla, CA, USA). All the statistics were carried out directly on the raw measurement results. The results of the semi-quantified immunolabeling and calculated ratios were evaluated by an unpaired *t*-test followed by a Mann–Whitney test to compare ranks. To analyze the DNA double-strand breaks and repair, we conducted two ANOVA, for the foci size comparison one-way ANOVA, and both were followed by Holm–Sidak’s multiple comparisons test. For the RT-qPCR results, we applied one-way ANOVA, followed by Tukey’s multiple comparisons test on our ΔCt values. The differences were considered significant as follows: * *p* ≤ 0.05; ** *p* ≤ 0.01; *** *p* ≤ 0.001; **** *p* ≤ 0.0001.

## 5. Conclusions

Thus, we can presume that the condition of the UC vessels and circulating fetal RBCs closely reflects the in vivo status of the fetus exposed to stressors. The significance of this work lies with the fact that these results can contribute to a better understanding of the molecular changes during any toxic exposure in general, and not limited to only the intrauterine development. In-depth knowledge of the pathways studied may identify additional therapeutic targets for the clinical and applied translational research in the near future.

## Figures and Tables

**Table 1:** Specific primer sequences designed for RT-qPCR reactions.

Gene	Orientation	Primer Sequence 5′→3′
*18s rrna*	Forward	GAAACGGCTACC ACATCCAAGG
	Reverse	CCGCTCCCAAGATCCAACTACG
*mmp2*	Forward	CGCTACGATGGAGGCGCTAA
	Reverse	CAGGTATTGCACTGCCAACTCTT
*mmp9*	Forward	CGCAGACATCGTCATCCAGT
	Reverse	AACCGAGTTGGAACCACGAC
*mt1e*	Forward	CATTCTGCTTTCCAACTGCCTG
	Reverse	GCAGCWCTTCTTGCAGGAGG
*mt2a*	Forward	CAACTGCTCCTGCGCCG
	Reverse	CAGCAGCTGCACTTGTCCG
*mt3*	Forward	CTCCTGCAAGTGCGAGGG
	Reverse	GCCTCAGCTGCCTCTCCG
*timp1*	Forward	TGTGAGGAATGCACAGTGTTT
	Reverse	CGGGACTGGAAGCCCTTTTC
*timp2*	Forward	ATGCAGATGTAGTGATCAGGGC
	Reverse	GGAGGGGGCCGTGTAGATA

**Table 2:** List of antibodies.

**Primary Antibodies**	**Host**	**Clonality**	**Dilution**	**Manufacturer**	**Reference**
4′, 6-diamidino-2-phenylindole (DAPI)	-	-	1:1000	Sigma-Aldrich	D9542
Anti-4-hydroxynonenal	mouse	mono	1:100	Abcam	ab48506
Anti-cleaved caspase-3	rabbit	poly	1:100	Abcam	ab2302
Anti-DNA PKcs (phospho S2056)	rabbit	poly	1:100	Abcam	ab18192
Anti-phospho-Histone H2A.X (Ser139)	mouse	mono	1:100	Sigma-Aldrich	05636i
Anti-CD235a (Glycophorin A)	mouse	mono	1:50	Thermo Fisher	MA5-12484
Anti-CD235a (Glycophorin A)	rabbit	mono	1:50	Invitrogen	PA5-115298
Anti-MMP-2 (4D3)	mouse	mono	1:100	Santa Cruz Biotechnology	sc-53630
Anti-MMP-9 (2C3)	mouse	mono	1:100	Santa Cruz Biotechnology	sc-21733
Anti-TIMP-1 (G-6)	mouse	mono	1:100	Santa Cruz Biotechnology	sc-365905
Anti-TIMP-2 (3A4)	mouse	mono	1:100	Santa Cruz Biotechnology	sc-21735
**Secondary Antibodies**	**Host**	**Clonality**	**Dilution**	**Manufacturer**	**Reference**
Anti-mouse IgG H&L (Alexa 647)	goat	poly	1:1000	Abcam	ab150115
Anti-rabbit IgG H&L (Alexa 488)	goat	poly	1:1000	Abcam	ab150077
Anti-mouse IgG H&L (Alexa 647)	goat	poly	1:1000	Abcam	ab150079
Anti-rabbit IgG H&L (Alexa 488)	goat	poly	1:1000	Abcam	ab150113

## Data Availability

Available upon request.

## Supplementary Materials

The supporting information can be downloaded at: https://www.mdpi.com/article/10.3390/ijms232314673/s1.
