# Supplementary material for: Molecular Background of Toxic-Substances-Induced Morphological Alterations in the Umbilical Cord Vessels and Fetal Red Blood Cells
Source: Int J Mol Sci. 2022 Nov 24;23(23):14673. doi: 10.3390/ijms232314673 (PMC9736329; doi:10.3390/ijms232314673)
Supplement: Supplementary file 1 [file ijms-23-14673-s001.zip › ijms-1959692-supplementary.pdf]

Supplementary figures

Molecular background of toxic substances induced morphological alterations in the umbilical cord vessels and fetal red blood cells

Szabolcs Zahorán[1,†], Ágnes Márton[1], Krisztina Dugmonits[1], Payal Chakraborty[1,‡], Ali Khamit[1], Péter Hegyi[2,3,4], Hajnalka Orvos[5] and Edit Hermesz [1,*]

[1] Department of Biochemistry and Molecular Biology, Faculty of Science and Informatics, University of Szeged, H-6701 Szeged, Hungary

[2] Institute for Translational Medicine, Medical School, University of Pécs, H-7601 Pécs, Hungary

[3] Centre for Translational Medicine, Semmelweis University, H-1085 Budapest, Hungary

[4] Division of Pancreatic Diseases, Heart and Vascular Center, Semmelweis University, H-1085 Budapest, Hungary

[5] Department of Obstetrics and Gynecology, Albert Szent-Györgyi Medical School, University of Szeged, H-6701 Szeged, Hungary

* Correspondence: hermesz@bio.u-szeged.hu; Tel.: +36-(62)-544-887

[†] Present address: Department of Microbiology and Molecular Medicine, Institute of Genetics and Genomics Geneva, Faculty of Medicine, University of Geneva, 1211 Geneva, Switzerland

[‡] Present address: Department of Pharmaceutical Technology, JIS University, 81, Nilgunj Road, Kolkata 700109, India.

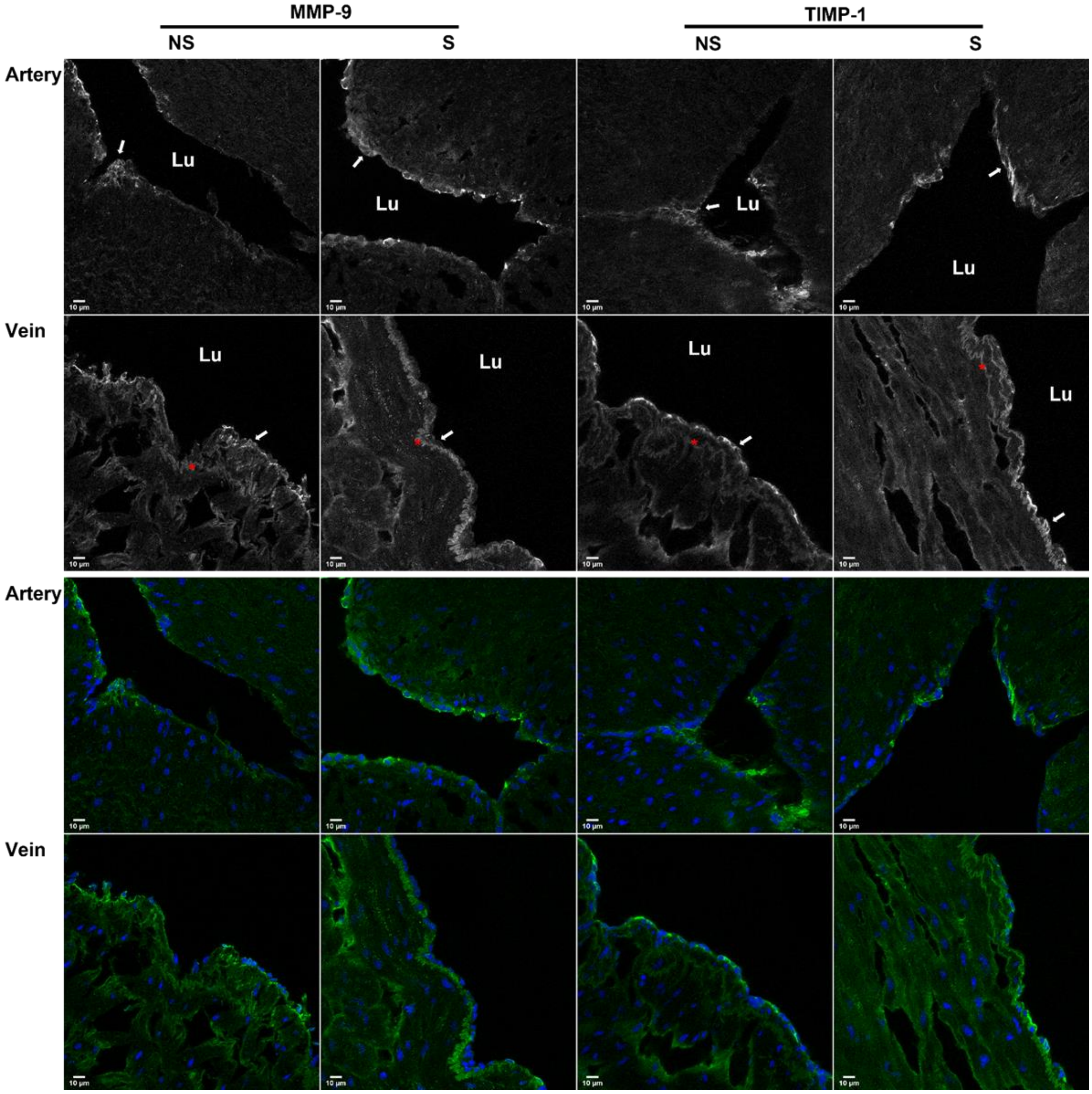

Figure S1. Representative confocal images of MMP-9 and TIMP-1 specific immunolabelling on the consecutive umbilical cord sections

Top two panels indicate the 8-bit images used for semi-quantification, corresponding to the TIMP-1 or MMP-9 channels, respectively. Bottom two panels are the merged DAPI, MMP-9/TIMP-1 images showing the exact localisation. White arrows – endothelial cells, Lu – vessel lumen, red asterisks – lamina basalis. Scale bar – 10 µm.

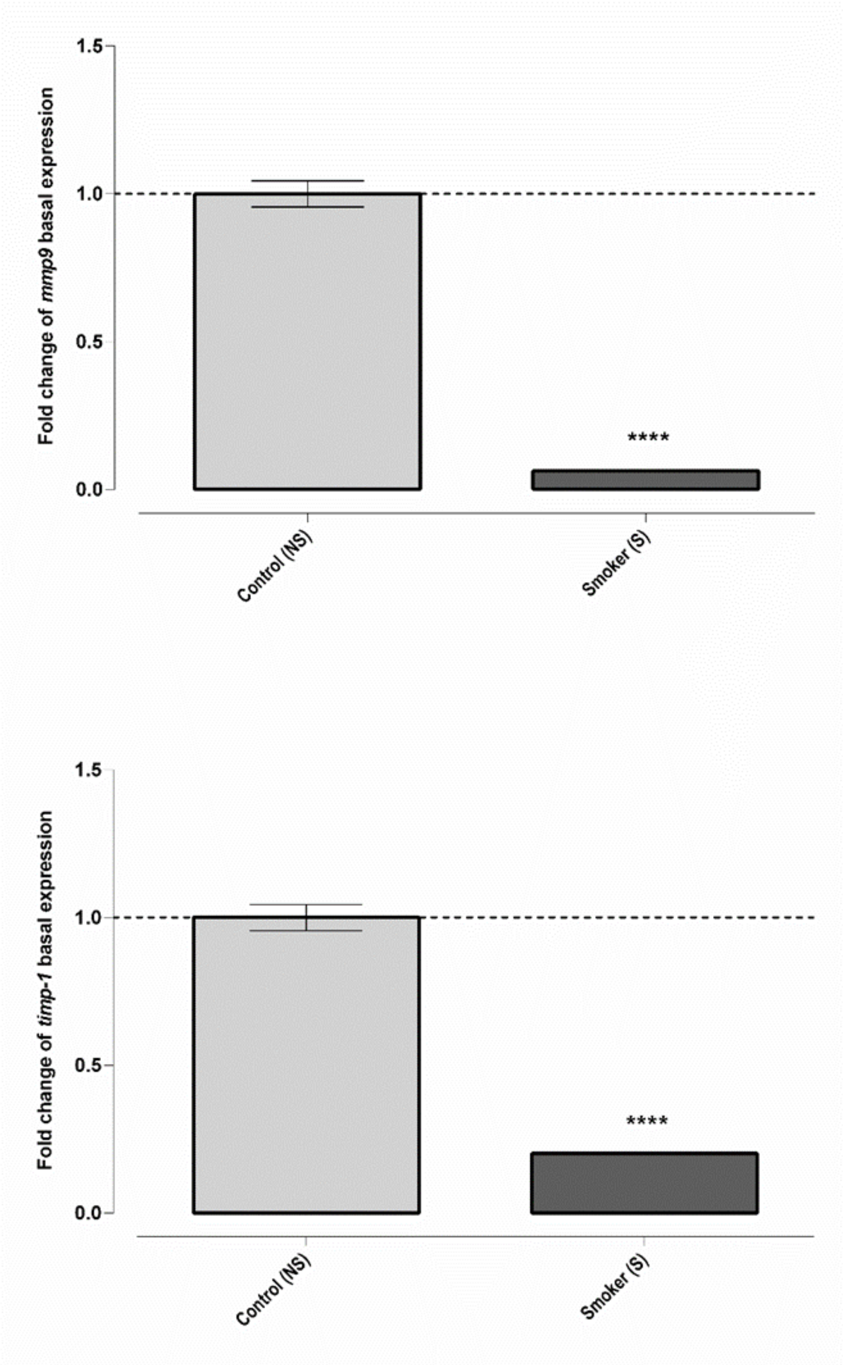

Figure S2. Differences in basal expression of *mmp-9* and *timp-1* genes in untreated Ns and S sample sets

Basal expression of *mmp-9* and *timp-1* genes in the samples with smoking origin relative to the untreated controls (dotted lines). Statistical analysis was done by using two-way (A) and one-way (B) ANOVA followed by Holm-Sidak's multiple comparisons test (**** $P \leq 0.0001$).

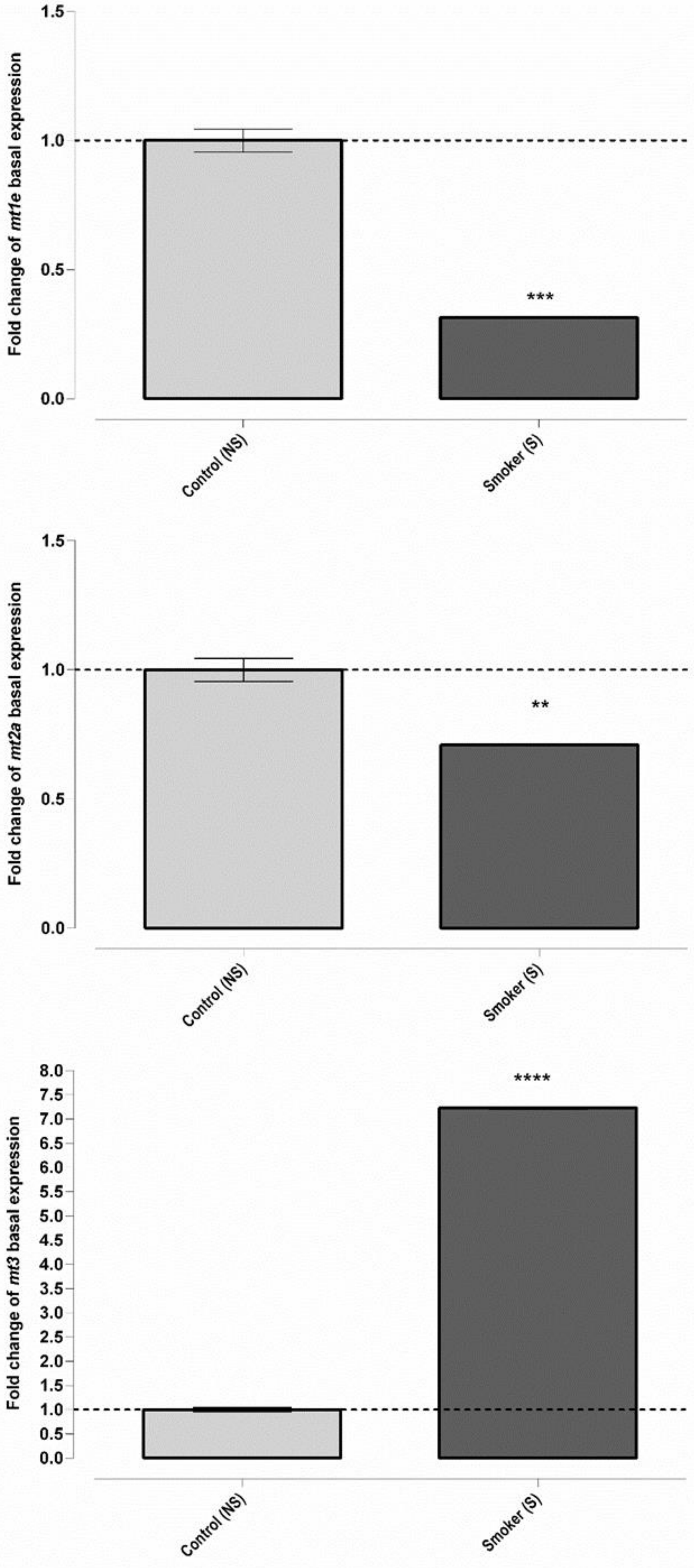
1.5
1.0
0.5
0.0
Fold change of *mt1e* basal expression
***
Control (NS)
Smoker (S)
1.5
1.0
0.5
0.0
Fold change of *mt2a* basal expression
**
Control (NS)
Smoker (S)
8.0
7.5
7.0
6.5
6.0
5.5
5.0
4.5
4.0
3.5
3.0
2.5
2.0
1.5
1.0
0.5
0.0
Fold change of *mt3* basal expression
****
Control (NS)
Smoker (S)

Figure S3. Differences in basal expression of metallothionein genes in the untreated Ns and S sample sets

Basal expression of *mt-1e*, *mt2a* and *mt3* genes in sample sets with smoking origin relative to the untreated controls (dotted lines). Statistical analysis was done by using two-way (A) and one-way (B) ANOVA followed by Holm-Sidak's multiple comparisons test (** $P \leq 0.01$; *** $P \leq 0.001$; **** $P \leq 0.0001$).
